# Thermodynamic efficiency, reversibility, and degree of coupling in energy conservation by the mitochondrial respiratory chain

**DOI:** 10.1038/s42003-020-01192-w

**Published:** 2020-08-18

**Authors:** Mårten Wikström, Roger Springett

**Affiliations:** 1 Institute of Biotechnology, University of Helsinki, Helsinki, Finland; 2 Cardiovascular Division, King’s College London, British Heart Foundation Centre of Excellence, 125 Coldharbour Lane, London, SE5 9NU UK

**Keywords:** Bioenergetics, Chemical biology

## Abstract

The protonmotive mitochondrial respiratory chain, comprising complexes I, III and IV, transduces free energy of the electron transfer reactions to an electrochemical proton gradient across the inner mitochondrial membrane. This gradient is used to drive synthesis of ATP and ion and metabolite transport. The efficiency of energy conversion is of interest from a physiological point of view, since the energy transduction mechanisms differ fundamentally between the three complexes. Here, we have chosen actively phosphorylating mitochondria as the focus of analysis. For all three complexes we find that the thermodynamic efficiency is about 80–90% and that the degree of coupling between the redox and proton translocation reactions is very high during active ATP synthesis. However, when net ATP synthesis stops at a high ATP/ADP^.^_Pi_ ratio, and mitochondria reach “State 4” with an elevated proton gradient, the degree of coupling drops substantially. The mechanistic cause and the physiological implications of this effect are discussed.

## Abstract (web summary)

Wikström and Springett analyze the thermodynamic efficiency of redox reactions and proton translocation by the complexes of mitochondrial respiratory chain. They report that the thermodynamic efficiency is about 80–90% and that the degree of coupling between the redox and proton translocation reactions is very high during active ATP synthesis, but decreases when ATP synthesis stops.

## Introduction

The mitochondria are the power plants of the eukaryotic cell. In most species, cell respiration is catalysed by a linear array of three energy-transducing membrane-bound proteins, viz. complexes I, III and IV. Each complex links the catalysed redox reaction (reduction of ubiquinone by NADH, reduction of ferricytochrome *c* by ubiquinol, and reduction of O_2_ by ferrocytochrome *c*, respectively) to net translocation of protons from the inside (mitochondrial matrix) to the outside (intermembrane space) of the inner mitochondrial membrane, thus creating an electrochemical proton gradient (protonmotive force, pmf, Δ*P*, Δ*μ*_H+_) as the primary form of conserved energy. Research over the last 40 years has revealed that the mechanisms of redox-linked proton translocation are fundamentally different for the three complexes^1–5^. Here, using specific sets of the published data with isolated mitochondria (see below), we address the tightness by which respiratory electron transfer in the three complexes is coupled to transmembrane proton translocation (the “degree of coupling”), and assess the thermodynamic efficiency of this process.

We find that during oxidative phosphorylation (State 3, ref. ^6^), when the Δ*P* is submaximal, the average thermodynamic efficiency is ca. 80–90% for all three complexes, and that the degree of coupling of proton translocation to electron transfer is extremely high. When the rate of respiration falls to a resting steady-state level due to a high ATP/ADP^.^P_i_ ratio (State 4), the degree of coupling is significantly lowered and free energy is dissipated as an increase in entropy. This has been ascribed to a proton leak across the inner mitochondrial membrane^7–10^, and/or to mechanistic “slipping” in the proton pumps themselves^11–13^. We find that the reduced degree of coupling in State 4 is not uniform among the three respiratory complexes, which suggests control at the mechanistic level of proton pumping.

## Results

### The approach

**Fig. 1:**
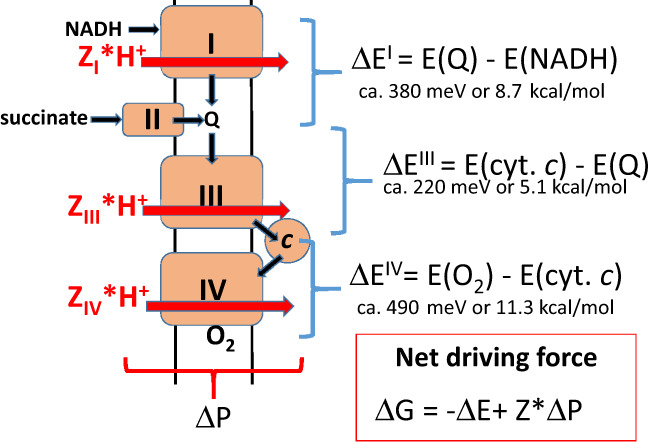
The mitochondrial respiratory chain. The protonmotive complexes I, III and IV are depicted in the inner membrane, together with additional components of the chain, complex II, cytochrome *c* and ubiquinone (Q), the hydrogen donors NADH and succinate and the acceptor, O_2_. The combination of N,N,N’,N’-tetramethyl-phenylene-diamine (TMPD) and ascorbate (not shown for clarity; see the text) donates electrons directly to cytochrome *c* and thus engages complex IV only. Black arrows depict the hydrogen and electron transfers in the chain, eventually reducing O_2_ to water. Red arrows symbolise the associated protonmotive events where *Z*_n_ is the effective protonmotive H^+^/e^−^ stoichiometry, 2 for complex I and complex IV, 1 for complex III (see “Methods”) and n is the name of the complex. Typical average redox potential differences (Δ*E*) across each complex are given. The net free energy change (Δ*G*) for any combination of complexes is given as the arithmetic sum of the relevant redox potential difference and the protonmotive force (Δ*P*) multiplied by the H^+^/e^−^ stoichiometry (*Z*), where Δ*E* has a negative sign and is larger than Δ*P* for the case where redox chemistry drives proton translocation.

Conceptually, all three proton-translocating complexes of the respiratory chain (Fig. 1) function in the same way, although the detailed molecular mechanisms are very different. In all cases, we are dealing with an energetically uphill, endergonic or “driven” reaction of proton translocation from the negatively charged N-side to the positively charged P-side of the inner mitochondrial membrane (defined here as reaction 1). Such a reaction cannot occur spontaneously, but becomes possible when coupled to an energetically downhill, exergonic or “driving” reaction (reaction 2), which is the oxidation of NADH by ubiquinone (catalysed by complex I), oxidation of ubiquinol by ferricytochrome *c* (catalysed by complex III) or oxidation of ferrocytochrome *c* by dioxygen (cataysed by complex IV), or any combination of these three functions (Fig. 1).

Treating this on the general level of thermodynamics of non-equilibrium coupled reactions, one may write (see refs. ^14–16^)

1a $$\begin{array}{*{20}{c}} {J_1} = {L_{11}X_1 + L_{12}X_2} \\ {J_2} = {L_{21}X_1 + L_{22}X_2} \end{array},$$

where *J*_*1*_ and *J*_*2*_ are the fluxes of the coupled reactions, and *X*_1_ and *X*_2_ refer to the corresponding forces (free energies). In the case of the electron-transport chain complexes, this becomes:

1b $$\begin{array}{*{20}{c}} {J_H} = { - L_{11}{\Delta}P - L_{12}{\Delta}E} \\ {J_e} = { - L_{21}{\Delta}P - L_{22}{\Delta}E} \end{array},$$

where *J*_*H*_ is proton flux (negative when protons are pumped from the N to the P-side of the membrane), *J*_*e*_ is the electron flux, Δ*P* is the protonmotive force and Δ*E* is the redox span or difference in redox potential of the redox carriers on the donor and acceptor side of the reaction considered. The cross-coupling coefficients *L*_12_ and *L*_21_ are assumed to be equal, so-called Onsager symmetry, which is met under many conditions of oxidative phosphorylation (see refs. ^15,16^ and below). For a pump, *L*_11_ and *L*_22_ are negative, and *L*_12_ and *L*_21_ are positive.

**Fig. 2:**
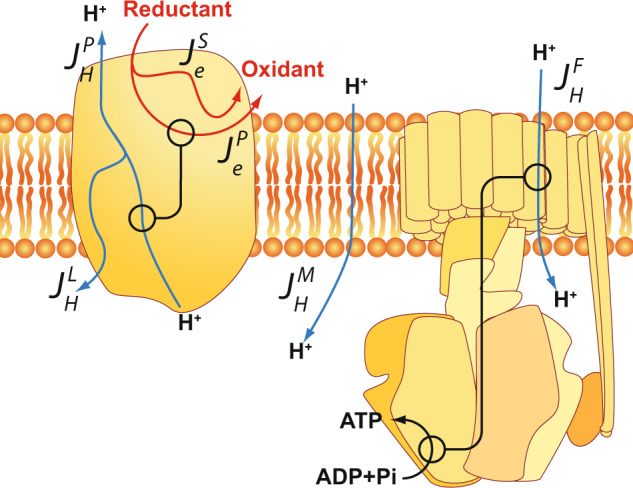
Leaks and slips in the protonmotive function of mitochondria. The cartoon shows a prototype of a protonmotive redox complex of the respiratory chain (left) and a schematic view of the F_1_F_o_ ATP synthase (right), both embedded in the inner mitochondrial phospholipid membrane. Red arrows depict electron transfer from reductant to oxidant, either linked (*J*_*e*_^*P*^) to proton pumping across the membrane (*J*_*H*_^*P*^) or decoupled from it (“electron slip”, *J*_*e*_^*S*^). *J*_*H*_^*L*^ depicts an intrinsic proton leak in the pump mechanism (“proton slip”). *J*_*H*_^*M*^ stands for loss of protonmotive force by a proton leak across the membrane. Proton influx across the F_o_ domain of the ATP synthase (*J*_*H*_^*F*^) is assumed to be fully coupled to synthesis of ATP without significant slips (see the text). The stoichiometric aspects (H^+^/e^−^ and H^+^/ATP ratios) are omitted for clarity (but see the text).

It is important to note that our treatment of mitochondrial redox-linked proton translocation by equations^1^ is a simplification insofar as the proton flux, *J*_*H*_, will be assessed from the proton flux across the F_o_ portion of the ATP synthase leading to ATP synthesis (see below). Equation (1b) does not explicitly include proton flux across the membrane dielectric (proton leak). Such proton leak, as well as incomplete coupling between electrons and protons in the respiratory complexes themselves (i.e. proton or electron “slipping”, see refs. ^7–13,17^ and Fig. 2), are instead collectively considered in the “degree of coupling” (*q*) of reactions 1 and 2, as will be defined below.

Defining the ratios of output and input forces and flows as *x* (=Δ*P*/Δ*E*) and *j* (=*J*_*H*_*/J*_*e*_), respectively, and the mechanistic H^+^/e^−^ stoichiometry, *Z*, as the square root of the ratio between the primary flow/force-coupling coefficients, i.e. *Z* = *√(L*_11_*/L*_22_*)*, one obtains from Eq. (1) ^14–16^

2 $$\frac{j}{Z} = \frac{{q - Zx}}{{Zxq - 1}},$$

where *q* is given by:

3 $$q = \frac{{L_{12}}}{{\sqrt {L_{11}L_{22}} }}.$$

The factor *q* has been called the degree of coupling between the input and output processes^14^ and varies between 0 (no coupling) and 1 (complete coupling). With complete coupling of the two reactions (i.e. *q* = 1), the flux ratio *j* is equal to *−Z*.

The ratio *j*/*Z*, equivalent to (H^+^/e^−^)/*Z*, has been called the “stoichiometric efficiency”^18^, which must not be confused with the thermodynamic efficiency (cf. below). *j*/*Z* is also not equivalent to the degree of coupling (*q*), except for the special case where *x* = 0, i.e. when the force of the driven reaction is zero (Δ*P* = 0) and the coupled reaction proceeds without a load (see Eq. (2)). This special case has been termed “level flow”^14–16^, and is a condition usually strived at in most methods of determining the H^+^/e^−^ ratio experimentally (but see below). It is noteworthy that such an experimentally derived H^+^/e^−^ ratio (*j*) at level flow is not equal to the true mechanistic stoichiometry (*Z*), but to that value multiplied by the degree of coupling, i.e. *qZ*.

### The focus

One aim of this work is to assess the thermodynamic efficiency of the redox-driven proton translocation. Since thermodynamic efficiency (*η*) is defined as the ratio between the output and input powers of two coupled reactions, and since power is the product of flux (*J*) and force (*X*) we may write

4 $$\eta = - J_H{\Delta}P/J_e{\Delta}E,$$

where the negative sign signifies that the driven reaction flux (*J*_*H*_) occurs in a direction against its conjugate force (Δ*P*), that is, the net proton flux is dominated by pumping rather than leak. From the earlier definitions of the flow and force ratios (*j* and *x*), this equation simplifies to

5 $$\eta = - jx.$$

**Fig. 3:**
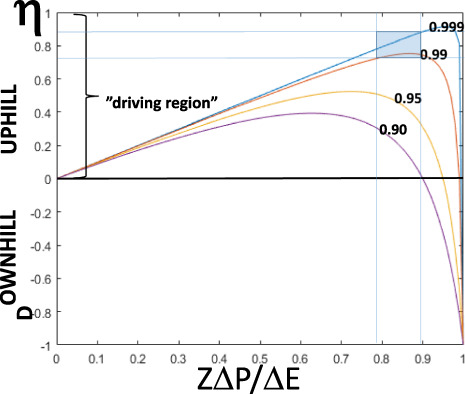
Theoretical dependence of thermodynamic efficiency on force ratio. The figure plots the dependence of efficiency (*η*) on the ratio between output (protonmotive) and input (oxidoreduction) forces normalised by the mechanistic (ideal) H^+^/e^−^ stoichiometry (*Z*) (see also ref. ^14^), according to Eq. (4), for four different degrees of coupling (*q*) as defined by Eq. (7). Example curves are given only for the “uphill” case where the redox reaction drives proton translocation. The thin blue lines show the approximate range of force ratios and efficiencies observed in State 3 of isolated rat-liver mitochondria (see Table 1). The area covered by the data is indicated as a blue rectangle.

Thus thermodynamic efficiency (*η*) of the whole-electron-transport chain approaches zero in State 4 mitochondria because the proton pumping is equal to the proton leak so that the net proton flux (*J*^*F*^_*H*_, Fig. 2) approaches zero. However, each individual proton-pumping complex could still be operating at high efficiency in State 4, but the energy transduced by the proton pumps is dissipated by leak through the membrane. In the fully uncoupled state, also called level flow^14–16^, the protonmotive force (i.e. the output energy, *X*_1_) approaches zero and hence *η* is zero as well. Clearly, the maximum efficiency of the overall reaction is achieved somewhere between static head and level flow (Fig. 3, for details see below). In a mathematical model of the proton pump of cytochrome *c* oxidase, Kim and Hummer^19^ found a very similar bell-shaped dependence of thermodynamic efficiency on the protonmotive force as the theoretical one shown in Fig. 3.

State 3, where excess of ADP and inorganic phosphate (P_i_) supports a high rate of respiration^6^ and concomitant synthesis of ATP driven by the Δ*P*, lies between static head and level flow and is hence the only reasonably well-defined state of isolated mitochondria suitable for determination of thermodynamic efficiency.

Determination of efficiency using Eq. (5) requires knowledge of the force ratio in State 3 (*x*), which can be calculated from literature data where the redox poise of the NAD^+^/NADH, ubiquinone/ubiquinol, ferri-/ferrocytochrome *c* and oxygen/water redox couples have been reported^20^. However, the flux ratio (*j*) (i.e. the H^+^/e^−^ ratio) cannot be directly measured in State 3. Fortunately, we have access to a meta-analysis of the amount of ATP formed per electrons transferred (the so-called ATP/2e^−^ or P/O ratio) in the different domains of the mitochondrial respiratory chain in State 3^21^. Such ATP/2e^−^ data can be converted to H^+^/2e^−^ ratios (which are twice the flux ratios, j) by the relation

6 $${\mathrm{H}}^ + /2{\mathrm{e}}^ - = {\mathrm{ATP}}/2{\mathrm{e}}^ - \times {\mathrm{H}}^ + /{\mathrm{ATP}}.$$

where H^+^/ATP, i.e. the number of protons translocated from the P-side to the N-side of the inner membrane per synthesised ATP molecule, is 3.67 in animal mitochondria for the case of extramitochondrially produced ATP. Due to the eight c subunits of the membrane ring structure of the ATP synthase in animal mitochondria, eight protons are translocated per full rotation with consequent synthesis of three molecules of ATP^22^, yielding 8/3 or 2.67 H^+^/ATP. Further translocation of the formed ATP to the extramitochondrial space, in exchange for ADP and P_i_ in the opposite direction, takes place on the ADP/ATP and phosphate carriers, respectively, in exchange for translocation of one more proton^22^.

It is essential to note that the flux ratio, *j*, assessed by this method, only measures the productive proton flux used for ATP synthesis (see Fig. 2 and below).

In order to derive the degree of coupling (*q*) in State 3 mitochondria, it is not sufficient to know the mechanistic stoichiometry (*Z*) and the H^+^/e^−^ ratio (*j*). As mentioned above, *q* = *j*/*Z* only in the special case where there is no load, i.e. where *x* = 0. State 3 mitochondria are not operating at level flow (Δ*P* = 0), but at a considerable force ratio (*x*); Δ*P* is often ca. 170 mV^7–10,13,23^. It is thus necessary to solve Eq. (3) with respect to *q*, yielding

7 $$q = \frac{{Zx - j/Z}}{{1 - jx}}.$$

Whilst the degree of coupling (*q*) can be considered a constant in this treatment, the H^+^/e^−^ ratio (i.e. the flux ratio *j*) is not, but decreases with increasing load (*x*) from its maximal value of *qZ* at zero load^14–16^.

### Thermodynamic efficiency

Table 1 shows the data obtained for the three redox complexes in isolated mitochondria in State 3, either operating together, or assessed separately (see “Methods”). For comparison between the three redox complexes, the force ratio (*x*) is normalised by multiplication with the mechanistic H^+^/e^−^ stoichiometry, *Z*, which is 2, 1 and 2, respectively for complexes I, III and IV (see “Methods”), and the flux ratio (*j*) is normalised by division with *Z*. The data for the individual complexes were obtained as follows (see Fig. 1). The ATP/2e^−^ for complex I was taken as the difference between the average ratios with NADH-linked substrates and succinate. The ratio for complex IV was based on the average ATP/2e^−^ with TMPD+ ascorbate as substrate. The ratio for complex III was based on the difference between ATP/2e^−^ ratios with succinate and TMPD+ ascorbate as substrates.

**Table 1:** Thermodynamic efficiency (*η*) and degree of proton–electron coupling (*q*) in State 3 of isolated rat-liver mitochondria.

	*Zx*	*j*/*Z*	*η*	*q*
Complexes I + III + IV	0.82 ± 0.00 (*n* = 3)	−0.97	79%	0.997
Complexes III + IV	0.82 ± 0.01 (*n* = 4)	−0.96	79%	0.996
Complex I	0.88 ± 0.01 (*n* = 3)	−0.98	87%	0.999
Complex III	0.80 ± 0.04 (*n* = 4)	−0.98	78%	0.998
Complex IV	0.81 ± 0.03 (*n* = 5)	−0.95	77%	0.995
(Complex IV)	0.70 ± 0.02 (*n* = 5)	−0.95	66%	0.991

*x* is the force ratio, between the protonmotive force (assumed at 170 mV) and the redox potential difference across the measured span of the respiratory chain. Extramitochondrial pH is 7.4; matrix pH = 8.0. Midpoint potentials at pH = 7.4 are 230 mV for cytochrome *c*, 66 mV for ubiquinone and 776 mV for O_2_/2H_2_O (at 120 μM O_2_). Midpoint potentials at pH = 8 are 30 mV for ubiquinone and −350 mV for NAD/NADH (see “Methods” and the text). *Z* is the mechanistic H^+^/e^−^ stoichiometry, which is 2, 1 and 2 for complexes I, III and IV, respectively (see “Methods”). *j* is the observed flux ratio (H^+^/e^−^). The values for complex IV as well as for complexes I + III + IV and III + IV are for the cases in which the energy of the irreversible splitting of the O–O bond has been subtracted and the concentration of O_2_ lowered to 12 μM (see the text). The value for complex IV in parenthesis is without subtraction of O–O bond splitting and at a mean O_2_ concentration of 120 μM. Values are expressed as mean ± SD, where *n* is the number of distinct samples.

As shown in Table 1, the calculated thermodynamic efficiency (*η*) varies between ~67 and 87%, the highest being the efficiency of complex I. Two sets of the data are listed for complex IV, which is explained in the next section.

### The special case of complex IV (cytochrome *c* oxidase)

**Fig. 4:**
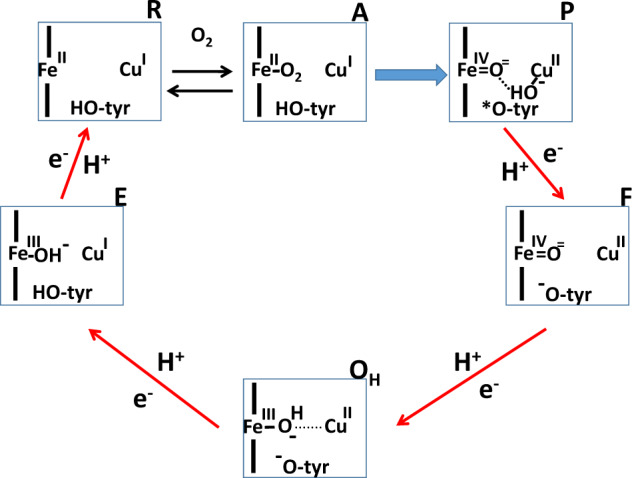
Catalytic intermediates of the binuclear centre of complex IV. The binuclear centre is shown as a rectangle, consisting of haem *a*_*3*_ (Fe) the nearby Cu_B_ (Cu) and the conserved tyrosine (tyr-OH, tyrosine; tyr-O^−^, tyrosinate; tyr-O*, neutral tyrosine radical) covalently linked to one of the three histidine ligands of Cu_B_. The three histidine ligands of Cu_B_ and the proximal histidine of haem *a*_*3*_ are not shown for clarity. The name of each intermediate state is found outside the right corner of each rectangle. The red arrows specify reactions that each involve electron transfer to the site from the low spin haem *a*, net transfer of one substrate proton from the N-side of the membrane to complete oxygen- reduction chemistry, and uptake (from the N-side) and release (to the P-side) of another proton (i.e. proton pumping). Hence, the red arrows indicate partial redox reactions that are reversible at high Δ*P*. The blue arrow depicts the irreversible partial reaction step where the O–O bond of O_2_ is broken in a reaction not linked to energy transduction. Note that the classical “ferrous-oxy” intermediate A^26^ is more accurate described as a ferric-superoxide state of haem *a*_*3*_^5^.

Conventional treatment of the complex IV data (Table 1, data within parentheses) indicates that the terminal enzyme of the respiratory chain would have by far the lowest energy transduction efficiency, 67%, as compared with the efficiencies of complex III (78%) and complex I (87%). One may suspect that this is related to the fact that the overall complex IV reaction is irreversible, so that generation of O_2_ from water is not possible as driven by the protonmotive force. However, elucidation of the mechanism of complex IV catalysis^1,24^ has revealed that the irreversibility is the result of an irreversible splitting of the O–O bond in the partial reaction step due to the large Δ*G*^0^, where the oxygen-bound intermediate A is converted into intermediate P (see ref. ^25^, Fig. 4), a reaction that is neither coupled to proton pumping nor to transmembrane electron or proton transfer^1,24^. The binding of O_2_ to intermediate R is fully reversible^26,27^, and most important, all the other four partial reactions of the catalytic cycle are coupled to proton translocation^1,24^, and have indeed been shown to be reversed by protonmotive force^28^.

The irreversibility of the overall cytochrome *c* oxidase reaction should therefore not be taken to suggest loose coupling of the redox chemistry to proton translocation. In order to focus analysis on these coupled reaction steps, we have subtracted the free energy change of the irreversible reaction step A → P (Fig. 4; ca. 5 kcal/mol^25^) from the overall standard driving force. In the basic analysis (Table 1, data in parentheses), we have assumed an average concentration of O_2_ of 120 μM in calculating the driving force. However, as shown by Wilson et al.^29^, the redox potential of the donor, cytochrome *c*, is not lowered (cyt. *c* is not getting more reduced) in the steady state until the oxygen concentration is lowered below ca. 10 μM. We have therefore adjusted the redox potential of the O_2_/2H_2_O couple to 12 μM O_2_ to obtain a better estimate of the true efficiency. As shown in Table 1, these two changes raise the thermodynamic efficiency from 67% to 77%, entirely due to the change in the force ratio. We conclude that the average thermodynamic efficiency of the actual proton pump elements of complex IV (red arrows in Fig. 4) is quite comparable to those of the other complexes, and that the lower overall efficiency of complex IV function is caused by the irreversible step of breaking the O–O bond that is not coupled to generation protonmotive force.

### The degree of coupling

Table 1 shows the results for the degree of coupling (*q*) calculated for State 3 according to Eq. (7). What is essential here is that the degree of coupling is very high for all three complexes of the respiratory chain; *q* is well over 0.99. Due to this, the normalised output/input force ratio (*Zx*) in State 3 yields a fair approximation of the thermodynamic efficiency (see Eqs. (2) and (5)), which gives credence to the use of this ratio as an efficiency indicator (e.g., ref. ^30^). The degree of coupling is fairly insensitive to the protonmotive force in State 3, which was assumed here to be 170 mV (see above). If the Δ*P* were higher, the degree of coupling would be even higher than in Table 1. If it were 150 mV, the degree of coupling for succinate oxidation (complexes III + IV), for example, would only fall to 0.992.

The very high degree of coupling (*q* > 0.99) found here from the data of phosphorylating (State 3) mitochondria might seem to be in sharp contrast to conclusions made earlier on the basis of the dependence of mitochondrial respiratory rates on membrane potential. For example, Hinkle et al.^23^ found that the rate of State 4 respiration, measured at the same membrane potential as in the phosphorylating State 3, was a considerable fraction of the rate of respiration in State 3. The energy loss assessed this way was almost 4% with succinate as substrate (complex III + IV activity) and ~7% for complex IV alone (see also “Discussion”). However, the flux ratios (H^+^/e^−^) in State 3 observed here were 0.95–0.98 after normalisation to *Z* (Table 1), suggesting a 2–5% loss to leaks which is consistent with the aforementioned data. It should be emphasised that as long as the degree of coupling is not ideal (i.e. *q* < 1), the flux ratio is bound to decrease with increasing force ratio^14–16^. As already concluded above, *j* is equal to *qZ* only at *x* = 0, i.e., at level flow when the protonmotive force is zero.

### The coupled reactions run close to equilibrium

Δ*G* denotes the free energy change of the coupled reaction of oxidoreduction-linked proton translocation for anyone of the three protonmotive complexes or their combination, i.e.

8 $$\begin{array}{*{20}{c}} {{\Delta}G} = { - {\Delta}E + Z{\Delta}P} \\ {} = {X_2 - ZX_1} \end{array}.$$

The redox span for the different complexes of the respiratory chain (X_2,_ or Δ*E*) may be obtained from the literature as before (Table 2^20,31^). For State 3, we have assumed a pmf of 170 mV (see above), and this yields a free energy change of ca. −44 mV for complexes I and III, which corresponds to ~−1 kcal/mol, showing that even in State 3 these complexes operate quite close to thermodynamic equilibrium. Complex IV appears less reversible, but when the irreversible O–O bond splitting is subtracted and the O_2_ concentration adjusted to physiological levels (see above), the average free energy change of the reactions that actually drive transmembrane proton transfer is only of the order of −1.8 kcal/mol (−78 mV).

**Table 2:** Driving force of the redox (Δ*E*) and of the overall redox-coupled proton translocation (Δ*G*) reactions in States 3 and 4.

	State 3		State 4		
	Δ*E* (mV)	Δ*G* (mV)	Δ*E* (mV)	Δ*G* (mV)	*q*
	(*X*_2_)	(*ZX*_1_ + *X*_2_)	(*X*_2_)	(*ZX*_1_ + *X*_2_)	
Complexes I + III + IV	1042 ± 5 (*n* = 3)	−192	1057 ± 9 (*n* = 3)	−123	0.880
Complexes III + IV	621 ± 4 (*n* = 3)	−111	643 ± 4 (*n* = 3)	−82	0.87
Complex I	384 ± 6 (*n* = 3)	−44	379 ± 4 (*n* = 3)	−5	>0.99
Complex III	214 ± 9 (*n* = 4)	−44	222 ± 6 (*n* = 4)	−35	0.84
Complex IV	418 ± 15 (*n* = 5)	−78	434 ± 22 (*n* = 5)	−60	0.86
(Complex IV)	488 ± 15 (*n* = 5)	−148	504 ± 22 (*n* = 5)	−131	

All numbers are in millivolts ± standard deviation. Δ*P* = 187 mV in State 4, 170 mV in State 3. The apparent *q* in State 4 is calculated according to Eq. (9). Complex IV data are calculated in two ways (see text and legend to Table 1). Values are expressed as mean ± SD, where *n* is the number of distinct samples.

The values for State 4 are also listed in Table 2. In the absence of an experimental value for Δ*P* in the Muraoka & Slater experiments^20^, we have estimated it as follows. We have collected the cases where respiration was studied with succinate and with TMPD + ascorbate as substrates^20^, where there is no or very limited net electron flux across complex I. Instead, the generated Δ*P* drives electrons backwards from cytochrome *c* and ubiquinone to NAD^+^ until a steady state is reached, in which the redox reactions of complex I should equilibrate with the Δ*P*. Under those conditions, we found that Δ*E* across complex I was 373 and 374 mV, respectively, i.e. corresponding to a Δ*P* of 187 mV, which seems to be a very good approximate for isolated rat-liver mitochondria in the presence of 10 mM inorganic phosphate^7,9^. Using this value for the Δ*P*, we find that complex I is indeed operating very close to thermodynamic equilibrium in State 4 during oxidation of NAD-linked substrates. By contrast, complexes III and IV deviate more from equilibrium (Table 2; see below).

### Is the degree of coupling a constant?

State 4 (static head) is defined as the state where net generation of Δ*P* (*J*_*1*_) vanishes. If *j* is zero in Eq. (2), then the numerator of the right hand side must be zero, from which follows that the force ratio, *x* = Δ*P*/Δ*E*, is a direct function of the degree of coupling^14–16^, such that

9 $$q = Zx.$$

This is a simplification because any proton leak across the membrane^7–10^ will ensure that net generation of Δ*P* (*J*_*1*_) by the pumping complexes still occurs at a rate determined by this leak. However, in well-coupled mitochondria the leak is slow relative to the forward and backward fluxes in the respiratory chain, so that the redox reactions of the respiratory complexes remain fairly close to equilibrium with the protonmotive force in State 4, and even in State 3 (Table 2).

Applying Eq. (9) to the State 4 data (Table 2) suggests that *q* is 0.99 for complex I, but only 0.84 and 0.90 for complexes III and IV, respectively, even though the latter is adjusted for the irreversibility of O–O bond splitting and assessed at 12 μM O_2_, as described above. Values of *q* significantly smaller than 0.99 have been reported before for isolated mitochondria^15^, but they have also been based on Eq. (9) and the State 4 situation.

This result suggests a close approach to thermodynamic equilibrium between the oxidoreduction reaction of complex I and its associated proton translocation in State 4, and thus that the degree of coupling is close to unity as it was found to be in State 3 (Table 1). By contrast, the degree of coupling for complexes III and IV appear to be clearly lowered from the value found in State 3, i.e., during active oxidative phoshorylation.

## Discussion

With regard to the requirement of Onsager symmetry in Eq. (1) in order to apply the current treatment, which is based on the principles of near-equilibrium irreversible thermodynamics^14^, such symmetry has been ensured experimentally for the reactions of oxidative phosphorylation^15,16,30,32^. In addition, it may be pointed out that under physiological conditions complexes I, III and IV turn over at rates that are very slow (see ref. ^30^) in comparison with the reaction rates of forward and backward electron and proton transfer within the three complexes. This means that the rates of physiological throughput are only a fraction of the individual forward and backward rates, again suggesting a system optimised to work close to thermodynamic equilibrium.

The thermodynamic efficiency of the three mitochondrial protonmotive redox complexes has not been systematically assessed thus far. Since thermodynamic efficiency is the product of the flux and force ratios of the output (H^+^ translocation) versus input (electron transfer) reactions (*η* = *−jx*; Eq. (5)), the flux ratio (*j*), which equals the H^+^/e^−^ ratio, is an essential parameter for such an assessment. A crucial obstacle has been that the thermodynamic efficiency approaches zero in the very experimental conditions strived at when measuring H^+^/e^−^ ratios, viz. conditions where the protonmotive force is zero, or very small (“level flow” condition). In the State 4 condition of maximal protonmotive force, there is an approach to zero net flux that also yields zero thermodynamic energy conservation efficiency. The individual pumping complexes themselves can operate at very high efficiency in State 4, but the overall efficiency of the electron-transport chain driving ATP synthesis is collapsed to zero due to the membrane leak (Fig. 2).

This problem can be approached by determination of the H^+^/e^−^ ratios of proton translocation by the mitochondrial respiratory chain in the phosphorylating State 3 from measured ATP/2e^−^ ratios, since the H^+^/ATP ratio of the F_1_F_o_ ATP synthase, and the translocation mechanisms of adenine nucleotides and inorganic phosphate are now known for animal mitochondria^22^. The implicit assumption here is that in State 3, these processes are “stoichiometric”, i.e., that there is no significant slippage in the mechanisms of the ATP synthase or the ADP/ATP and inorganic phosphate carriers. Thus the overall ratio of protons translocated per ATP synthesised extramitochondrially is assumed to be 3.67 (Eq. (6)). Some data^33^ suggested that the actual H^+^/ATP ratio may be somewhat lower than the one predicted from the structure of the F_o_F_1_ complex, but a later very careful account has suggested that the ratio is precisely determined by the structure^34^. If, nevertheless, the H^+^/ATP ratio were lower, the thermodynamic efficiencies reported here would be somewhat lower. More important, if that were the case, the estimates of the degree of coupling would have to be adjusted upwards, which seems unlikely considering that *q* is already higher than 0.99.

State 3 is indeed the only formally relevant mitochondrial condition for determining the thermodynamic efficiency of energy transduction, and was found here to be ca. 75–90% overall (Table 1). As shown in Fig. 3, such high efficiencies combined with the observed range of force ratios require a degree of coupling of at least 0.99. Rocha and Springett^30^ reported thermodynamic efficiencies for complexes I and III in intact isolated cells exceeding 90%, but these were based on the force ratio alone, corresponding to *Zx* in Table 1. Hence they relied on the assumption that the flux ratio equals the mechanistic stoichiometry, and therefore that *q* = 1. As shown in Table 1, this is a reasonable approximation for isolated mitochondria. The higher efficiency observed in cells compared with mitochondria could well be due to the fact that the cells were not studied under true State 3 conditions but under more natural conditions of energy load, which is expected to affect the efficiency^16^.

Correcting the experimentally obtained H^+^/e^−^ values for the force ratio in State 3 made it possible to determine the degree of coupling (*q*), which was found to be extremely high for all three complexes. In conditions extrapolated to level flow where Δ*P* = 0, Eq. (10) applies, viz.

10 $$j = - qZ.$$

Hence, our finding that *q* ≥ 0.99 means that the flux ratio (*j*) at level flow is very closely equal to the mechanistic H^+^/e^−^ stoichiometry (*Z*), which in turn implies that despite the full redox-driving force, there can be little or no leakage due to “uncoupled” electron transfer (“electron leak”).

These findings also corroborate earlier independent conclusions^8–10,13,23^ that slip and leak in the proton pumps of the respiratory chain do not occur significantly in mitochondria during oxidative phosphorylation. A slip in proton pumping is defined as a decrease of the H^+^/e^−^ stoichiometry either due to an electron transfer step being possible without linkage to protons (“electron slip”, excluded above for State 3), and pump leak is defined as the possibility of an already translocated proton being able to leak back to its original location by a failure of the pump mechanism (“proton slip”), or by a proton leak of the membrane (see Fig. 2). The latter two cases of proton leak, driven by Δ*P*, were found to occur to a total maximum of ca. 5% of the total flux in State 3.

In State 4 mitochondria (static head in the nomenclature of the theory of irreversible thermodynamics), Δ*P* is maximised. It is often assumed that there is no net proton pumping and that a near-equilibrium state is reached between the redox and proton translocation reactions. Such a near-equilibrium in State 4 was already concluded by Muraoka and Slater in their seminal work from 1969^35^. Although, in reality, a membrane proton leak balances redox-linked proton translocation by the three respiratory chain complexes, this leak is much slower than the forward and backward reactions of redox-linked proton translocation ensuring that a state near-equilibrium is reached. It is well known from the classical work by Nicholls^7^, Hinkle^23^ and Brand^8–10^ that when the protonmotive force increases beyond ca. 170 mV, the initially linear (Ohmic) dependence of membrane proton conductance on Δ*P* becomes exponential and the dependence is much steeper. This general proton leak (Fig. 2), the physiological importance of which has been stressed, has been ascribed to be an unspecific property of the mitochondrial solute carriers of the inner mitochondrial membrane, of which the ADP/ATP carrier is the most abundant^36^.

It is therefore not surprising that when the degree of coupling (*q*) is assessed based on Eq. (9), i.e. assuming that redox-linked proton translocation is zero (*j* = *0*), it is found to be lowered from the values observed in State 3 (see Table 2). However, if the flux in State 4 is at least predominantly due to membrane proton leak, then the flux ratio (*j*) cannot be assumed to be zero, but is a function of *q*, *Z* and *x* according to Eq. (2). Interestingly, in State 4 the driving force (Δ*G*) for complex I is very low (Table 2), showing that the redox-driven proton translocation is very close to thermodynamic equilibrium (cf above). However, complexes III and IV appear to remain further from equilibrium (Table 2). It thus seems that in State 4 whilst complex I approaches equilibrium closely, complexes III and IV remain further away. It may be especially revealing that the overall driving forces (ΔG) across complexes I and III are similar in State 3, but that upon the rise of Δ*P* to State 4 values, complex I comes very near to equilibrium, whereas complex III does not. We emphasise that the high absolute value of *q* that we find for complex I in State 4 (Table 2) is not in discord with the fact that there is a significant membrane leak under such conditions^7–10^. The high State 4 value of *q* in Table 2 is due to the simplification by treating State 4 according to Eq. (9), where it is assumed that net proton flux (*J*_*1*_ in Eq. (1a), or *J*_*H*_^*F*^ in Fig. 2) is zero, when in actual fact it constitutes the sum of the membrane proton leak (*J*_*H*_^*M*^; Fig. 2) and possible slips in the pumps (*J*_*H*_^*L*^; Fig. 2).

It is the observation of a different behaviour of complexes III and IV relative to complex I in State 4 that is difficult to explain on the basis of a membrane proton leak, which should affect all three complexes similarly, and this suggests that slips between the oxidoreduction and proton translocation reactions may occur in complexes III and IV driven specifically by the high Δ*P* in that state.

Experiments by Wilson et al.^37^ have shown that even a small lowering of the energy state (here considered to be the protonmotive force) can compensate for the lowered rate of respiration due to a decrease in oxygen tension. Thus local hypoxia will increase the level of reduced cytochrome *c*, which causes compensatory enhancement of the turnover of cytochrome *c* oxidase, and hence of the entire respiratory chain. This modulation will be all the more efficient if the linkage to proton pumping is loosened at high protonmotive force, which would otherwise prevent the compensatory increase in turnover. Interestingly, a model study by Krab et al.^38^ also showed that a decrease in protonmotive force speeds up the respiratory rate at low O_2_ concentrations, which was observed as a decrease in the apparent K_M_ for O_2_. Clearly, the terminal member of the respiratory chain, complex IV, is a very sensitive detector of tissue anoxia. The departure from equilibrium and partial loss of coupling efficiency of complex IV in State 4 (Table 2) is indicative of an important role in metabolic regulation^39–41^. The presence of “pulsed” and “resting” forms of complex IV^42,43^ might well be examples of how its energy transduction efficiency may be modulated.

We conclude that the thermodynamic efficiency of the three protonmotive respiratory chain complexes is ca. 80% during oxidative phosphorylation in State 3 of isolated rat-liver mitochondria. Under the same conditions, the degree of coupling between redox activity and proton translocation is more than 99% for all three complexes. However, at a high enough ATP/ADP^.^P_i_ ratio, when net phosphorylation stops and the respiratory rate reaches a minimum (State 4), the degree of coupling is lowered significantly more for complexes III and IV than it is for complex I. Although it is clear that a membrane proton leak is also present in State 4, the different effect on complexes III and IV relative to complex I suggest significant “slipping” of the proton pump machineries of the two former complexes at high protonmotive force. Such slipping is likely to be of physiological importance for at least three reasons. First, it counteracts a blockade of respiratory flux at high energy conditions. Secondly, it minimises accumulation of highly reduced respiratory intermediates and hence their bimolecular reaction with oxygen, producing superoxide. Thirdly, it modulates the reaction of complex IV at low concentrations of oxygen in such a way as to raise the apparent oxygen affinity, an additional security measure that maintains respiratory flux in hypoxia. Finally, our observations suggest that metabolic control mechanisms of cell respiration should primarily be sought from the functions of complexes III and, in particular, complex IV.

## Methods

### Experimental data

The basic experimental data (redox states of NAD^+^, ubiquinone (Q) and cytochrome *c*) are derived from the work by Muraoka and Slater^20^, who studied isolated rat-liver mitochondria respiring either on one of three NAD-linked substrates, β-hydroxybutyrate, pyruvate and glutamate, on succinate, or on TMPD + ascorbate, at pH = 7.4. The pH in the mitochondrial matrix was assumed to be 8^23^. Under such conditions, the Δ*P* in State 4 is estimated to be ca. 187 mV (see “Results”), and is lowered to ca. 170 mV in State 3 (see refs. ^7,23^). The redox potentials of the NAD^+^/NADH, ubiquinone/ubiquinol (Q/QH_2_), and ferric/ferrous cytochrome *c* couples were calculated from the measured extent of reduction^20^ using the Nernst equation, and the appropriate midpoint potentials (*E*_m_) relative to the Normal hydrogen electrode (see also Supplementary Data). For complex I, we used the matrix pH of 8.0 to calculate the redox potentials of NAD^+^/NADH and Q/QH_2_ (*E*_m,8_ values of −350 mV and +30 mV, respectively). For complex III, the midpoint redox potential of the donor, Q/QH_2_, was taken to be an *E*_m,7.4_ of +66 mV (note, using the extramitochondrial pH), and for the acceptor (cytochromes *c* + *c*_*1*_) we used +230 mV due to the low ionic strength of the reaction medium.

For complex IV, we used the *E*_m,7.4_ = +230 mV for the donor, as before. For the acceptor, the O_2_/2H_2_O redox couple, we used the extramitochondrial pH (=7.4) as the reference point (see below). The *E*_m,7_ of the O_2_/2H_2_O couple is 815 mV at 1 atm fugacity and 25 °C, corresponding approximately to 1.2 mM of O_2_. In the conventional assessments of the redox potential across complex IV, we have assumed that [O_2_] = 120 μM, i.e., at about half-way of the oxygen concentration scale in experiments with isolated mitochondria suspended in air-equilibrated buffer at 25 °C. In the special assessments of the force ratio across the proton-pumping reactions of complex IV (marked separately in Tables 1 and 2), we have adjusted the force ratio (and the other parameters) in two ways. First, we have taken into account that one of the partial catalytic reactions of complex IV catalysis is an “irreversible” scission of the O–O bond of O_2_ that is not coupled to the proton pump (Fig. 4). This roughly amounts to a 5 kcal/mol^25^ (~220 mV) subtraction of the free energy expenditure in the overall O_2_ reduction cycle. Secondly, as the redox potential of cytochrome *c* does not shift towards a more negative value until the oxygen concentration falls below ca. 10 μM^29^, we have used an O_2_ concentration of 12 μM.

### Thermodynamic H^+^/e^−^ stoichiometries

The reason for using the mitochondrial matrix pH for the donor and acceptor potentials of complex I but the extramitochondrial pH for the potentials of complexes III and IV is explained below. We also address the often confusing thermodynamic H^+^/e^−^ stoichiometries of 2, 1 and 2 for complexes I, III and IV, when the number of protons released (per electron) on the cytoplasmic side of the membrane is different, viz. 2, 2 and 1, respectively. Note, however, that the number of protons taken up from the matrix side coincides with the thermodynamic stoichiometries. For complex I, one proton per electron is taken up from the matrix on reduction of ubiquinone, and one is released back to the matrix on oxidation of the NAD-dependent substrate. Hence, the net substrate proton “count” is zero, even though oxidoreduction of NADH is linked to only 0.5 H^+^/e^−^ at a pH near 7.

Redox energy is transduced into a pH gradient (ΔpH) when protons are taken up from a low proton concentration (matrix side, high pH) and released into a high proton concentration (cytosolic side, low pH). The amount of energy conserved is *n*_*H*_*×*ΔpH, where *n*_*H*_ is the number of protons taken up and released. In contrast, redox energy is transferred into ΔΨ when charge moves across the membrane. The energy conserved is *n*_*Q*_ *×* ΔΨ, where *n*_*Q*_ is the number of translocated charges. The protons taken up or released in the reduction or oxidation of NADH, ubiquinone, or oxygen are “substrate protons” that should basically not be included in the calculation of energy conservation, as they are already included in their redox potentials (but see below, and ref. ^30^).

For a pure proton pump, such as complex I, the number of protons taken up and released is equal to the number of charges transferred across the membrane and the energy conserved is *n*_*P*_*(*ΔpH + ΔΨ*)* = *n*_*P*_Δ*P*, where *n*_*P*_ is the number of protons pumped. The Δ*G* (per electron) of the reaction catalysed by complex I (Δ*G*^*I*^*)* is thus:

11 $${\Delta}G^I = - \left( {E_h^{{\mathrm{UQ}}} - E_h^{{\mathrm{NADH}}}} \right) + 2{\Delta}P,$$

where *E*_*h*_^UQ^ and *E*_*h*_^NADH^ are the redox potentials of the ubiquinone and NADH pools using the pH in the matrix.

Complex IV pumps one proton per electron, which conserves one *Δ*P of redox energy. In addition, the electron starts on the cytosolic side and moves to the binuclear centre buried in the membrane, and a substrate proton moves from the matrix side to the binuclear centre to complete O_2_ reduction to water. The net effect is to move an additional positive charge across the entire membrane, conserving another ΔΨ of redox energy, giving a total energy conservation of Δ*P* + ΔΨ. The Δ*G* for complex IV is (Δ*G*^*IV*^) is then:

12 $${\Delta}G^{IV} = - \left( {E_h^{O,m} - E_h^{{\mathrm{Cyt}}c}} \right) + {\Delta}P + {\Delta}{\Psi},$$

where *E*_*h*_^*O,m*^ is the redox potential of the O_2_/H_2_O couple based on the matrix pH, and *E*_*h*_^Cyt*c*^ is the (pH-independent) redox potential of Cyt*c*. However, if the redox potential of the O_2_/H_2_O couple is instead calculated based on the cytosolic pH (*E*_*h*_^*O,c*^), Eq. (12) becomes:

13 $${\Delta}G^{IV} = - \left( {E_h^{O,c} - E_h^{{\mathrm{Cyt}}c}} \right) + 2{\Delta}P.$$

The situation for complex III is more complicated: for each electron transferred to cytochrome *c*, two protons are released from the Q_o_ centre to the cytosolic side and one proton is taken up from the matrix at the Q_i_ centre. However, this does not result in charge movement across the membrane because the Q_o_ centre is on the cytosolic and the Q_i_ centre on the matrix side of the membrane. Instead, one electron moves across the membrane from the Q_o_ to the Q_i_ centre (via the *b* cytochromes), which is equivalent to one positive charge moving from the matrix to the cytosolic side, and so conserves one ΔΨ of redox energy. The release of protons to the cytosolic side and uptake of protons from the matrix are not basically included in the energy conservation calculation because they are substrate protons of the oxidoreduction of ubiquinone, and hence included in the redox potential of UQ/UQH_2_. However, the redox potential of UQ/UQH_2_ at the Q_o_ site is different from that at the Q_i_ site for the same UQ/UQH_2_ ratio, because the sites use protons from different compartments at different concentrations. Mechanistically, one electron from the oxidation of UQH_2_ at Q_o_ reduces Cyt*c* and one reduces UQ at the Q_i_ site. The Δ*G* for complex III (Δ*G*^*III*^) is then

14 $${\Delta}G^{III} = - \left( {E_h^{{\mathrm{Cyt}}c} + E_h^{{\mathrm{UQi}}} - 2E_h^{{\mathrm{UQo}}}} \right) + {\Delta}{\Psi},$$

where *E*_*h*_^UQi^ and *E*_*h*_^UQo^ are the redox potentials of the UQ/UQH_2_ pools at the Q_i_ and Q_o_ sites, respectively. The difference between *E*_*h*_^UQo^ and *E*_*h*_^UQi^ is ΔpH (in millivolts) (see supplementary data of ref. ^44^), so that Eq. (14) can be rewritten as:

15 $${\Delta}G^{III} = - \left( {E_h^{{\mathrm{Cyt}}c} - E_h^{{\mathrm{UQo}}}} \right) + {\Delta}P.$$

Thus, as shown by Eqs. (13) and (15), using the pH of the cytosol to calculate the Δ*G* of complexes III and IV simplifies the thermodynamic analysis.

### Statistics and reproducibility

The P/O ratios required to calculate flux ratios in State 3 (Eq. (6); Table 1) were mean values taken from ref. ^21^, omitting the data published before 1960 due to its very high individual variability (Supplementary Tables 1 and 2). Thus *n*, the number of measurements, was 9, 14 and 5, respectively, for the spans of complexes I + III + IV, III + IV and IV. The redox spans required to determine force ratios (*x*) in Tables 1 and 2 were obtained from ref. ^20^. All the available data were used, i.e., respiration on β-hydroxybutyrate, pyruvate and glutamate (complexes I + III + IV), succinate (complexes III + IV) and ascorbate + TMPD (N,N,N’,N’-tetramethyl-p-phenylene diamine; complex IV). The results were taken as the mean ± SD with *n* values of 3 for complex I, 4 for complex III and 5 for complex IV (Supplementary Table 3).

### Reporting summary

Further information on research design is available in the Nature Research Reporting Summary linked to this article.

## Supplementary information

Supplementary Information

Supplementary Data 1

Description of Additional Supplementary Files

Reporting Summary

## Data Availability

All data supporting the findings of this study are available within the paper and its Supplementary Files or available from the corresponding author upon reasonable request. Oxidation states of NAD^+^, UQ and Cyt*c* and P/O ratios that support the findings of this paper were taken from Muraoka and Slater^20^ and Hinkle^21^, respectively, and reproduced in Supplementary Tables 1–3. The redox potentials and thermodynamic parameters derived from these data are given in the Supplementary Data file.
