# Supplementary material for: Thermodynamic efficiency, reversibility, and degree of coupling in energy conservation by the mitochondrial respiratory chain
Source: Commun Biol. 2020 Aug 18;3:451. doi: 10.1038/s42003-020-01192-w (PMC7434914; doi:10.1038/s42003-020-01192-w)
Supplement: Supplementary file 1 — Supplementary Information [file 42003_2020_1192_MOESM1_ESM.pdf]

P/O ratios

The P/O rations were taken from Hinkle (21) and only data from 1960 forward was used as there was too much variation in the pre-1960 data. The data used in the manuscript is given in supplementary table 1

	NADH-O_2	Succ-O_2	Site 3
Jacobs and Sanadi 1960			0.95
Estabrook 1967		1.7	
Lee et al. 1967			
Chamalaun and Tager 1969			0.94
Klingenberg 1975		1.4	
Hinkle and Yu 1979	2.2	1.4	
Pozzan et al. 1979			
Azzone et al. 1979		1.9	1.3
Lemasters 1984	2.6	1.7	
Beavis and Lehninger 1986	2.7–2.9	1.6–1.8	
Jensen et al. 1986		1.4–1.8	
Stoner 1987	2.6	1.5	1.0
Hafner and Brand 1988		1.4	
Luvisetto and Azzone 1989		1.3–1.5	
Toth et al. 1990	3.3–3.5		
Hinkle et al. 1991	2.3	1.5	0.98
Davis and Davis-von Thienen 1991	2.5		
Lee et al. 1996	2.9	1.8	
Fontaine et al. 1997		1.3–1.5	
Devin et al. 1997	2.5		
Gnaiger et al. 2000		1.6	
Mean±SD (n)	2.64±1.2 (9)	1.57±0.05 (14)	1.03±0.07 (5)

Supplementary table 1. P/O ratios from Hinkle (21) used in the manuscript.

Calculation of J or H^+/e^-

The proton to electron ratio (H^+/e^-), which is equal to *j*, is calculated from equation 6 of the manuscript using the P/O ratios of table S1. The calculation of the P/O ratios of complex I and III from the values in supplementary table 1 is given in the methods. The calculated values are given in supplementary table 2 along with *j/Z*.

	H^+/e^-	*j/Z*
Complex I+III+IV	4.84	0.968
Complex III+IV	2.87	0.957
Complex I	1.97	0.985
Complex III	0.98	0.98
Complex IV	1.89	0.945

Supplementary table 2. H^+/e^- ratio and j/Z calculated using the P/O ratios of supplementary table 1.

Redox potentials in State 3 and State 4

The reduction states of NADH, Ubiquinone and cytochrome c in state-3 and state 4 were digitized from the TMPD, succinate, β-hydroxybutyrate, pyruvate, glutamate data from figures 2, 3, 5,6 and 7, respectively, of Muraoka & Slater (20) and given in supplementary table 3.

	State 4			State 3		
Substrate	NADH	UQ	Cytc	NADH	UQ	Cytc
Glutamate	54	56	12	22	24	12
Pyruvate	35	46	8	19	14	5
B-OH Butyrate	67	62	9	33	19	6
Succinate		90	28		59	11
TMPD			38			18

Supplementary table 3. Values of the reduction state of NADH, ubiquinone (UQ) and cytochrome c (Cytc) with different substrates, expressed as % reduced used in the manuscript.

These reduction states were used in the supplementary spreadsheet to calculate the redox potentials and other values of table 2 of the manuscript.

The values of Zx from table I of the manuscript are also calculated in the spreadsheet. The value of j/Z of table 1 is calculated in supplementary table 2 and the values of η and q are calculated using equations 5 and 7 of the manuscript.
