# Supplementary material for: Thermodynamic efficiency, reversibility, and degree of coupling in energy conservation by the mitochondrial respiratory chain
Source: Commun Biol. 2020 Aug 18;3:451. doi: 10.1038/s42003-020-01192-w (PMC7434914; doi:10.1038/s42003-020-01192-w)
Supplement: Supplementary file 3 — Description of Additional Supplementary Files [file 42003_2020_1192_MOESM3_ESM.pdf]

Description of Additional Supplementary Files

File Name: **Supplementary Data 1**
Description source data file
